# Lipid nanoparticle-encapsulated mRNA antibody provides long-term protection against SARS-CoV-2 in mice and hamsters

**DOI:** 10.1038/s41422-022-00630-0

**Published:** 2022-02-24

**Authors:** Yong-Qiang Deng, Na-Na Zhang, Yi-Fei Zhang, Xia Zhong, Sue Xu, Hong-Ying Qiu, Tie-Cheng Wang, Hui Zhao, Chao Zhou, Shu-Long Zu, Qi Chen, Tian-Shu Cao, Qing Ye, Hang Chi, Xiang-Hui Duan, Dan-Dan Lin, Xiao-Jing Zhang, Liang-Zhi Xie, Yu-Wei Gao, Bo Ying, Cheng-Feng Qin

**Affiliations:** 1 State Key Laboratory of Pathogen and Biosecurity, Beijing Institute of Microbiology and Epidemiology, AMMS, Beijing, China; 2 School of Medicine, Tsinghua University, Beijing, China; 3 Kunming University of Science and Technology, Kunming, Yunnan China; 4 Suzhou Abogen Biosciences Co., Ltd, Suzhou, Jiangsu China; 5 Key laboratory of Jilin Province for Zoonosis Prevention and Control, Changchun, Jilin, China; 6 Beijing Protein and Antibody R&D Engineering Center, Sinocelltech Ltd, Beijing, China

**Keywords:** Biological techniques, Immunology

## Abstract

Monoclonal antibodies represent important weapons in our arsenal to against the COVID-19 pandemic. However, this potential is severely limited by the time-consuming process of developing effective antibodies and the relative high cost of manufacturing. Herein, we present a rapid and cost-effective lipid nanoparticle (LNP) encapsulated-mRNA platform for in vivo delivery of SARS-CoV-2 neutralization antibodies. Two mRNAs encoding the light and heavy chains of a potent SARS-CoV-2 neutralizing antibody HB27, which is currently being evaluated in clinical trials, were encapsulated into clinical grade LNP formulations (named as mRNA-HB27-LNP). In vivo characterization demonstrated that intravenous administration of mRNA-HB27-LNP in mice resulted in a longer circulating half-life compared with the original HB27 antibody in protein format. More importantly, a single prophylactic administration of mRNA-HB27-LNP provided protection against SARS-CoV-2 challenge in mice at 1, 7 and even 63 days post administration. In a close contact transmission model, prophylactic administration of mRNA-HB27-LNP prevented SARS-CoV-2 infection between hamsters in a dose-dependent manner. Overall, our results demonstrate a superior long-term protection against SARS-CoV-2 conferred by a single administration of this unique mRNA antibody, highlighting the potential of this universal platform for antibody-based disease prevention and therapy against COVID-19 as well as a variety of other infectious diseases.

## Introduction

Coronavirus disease 2019 (COVID-19) caused by the newly identified severe acute respiratory syndrome coronavirus 2 (SARS-CoV-2), has resulted in a public health crisis worldwide.^1^ As of January 3, 2022, there are 290,439,443 confirmed cases and 5,459,800 deaths worldwide, with 220 countries/regions affected (https://coronavirus.jhu.edu/map.html). Global efforts are ongoing to treat COVID-19 and to flatten the pandemic curve. To date, several vaccines, including inactivated vaccine, recombinant protein, adenovirus vector, and mRNA vaccine have been approved for the prevention and control of pandemic.^2^ However, only a few antiviral drugs have shown therapeutic effect in clinical trials,^3^ and the development of safe and effective countermeasures remains of high priority.

While vaccination represents the best strategy to prevent COVID-19, neutralizing monoclonal antibodies (mAbs)-based therapy could still benefit people before or after exposure to SARS-CoV-2, especially to those who haven’t received vaccines or who just received vaccines but before neutralizing titers could reach the protection threshold. To date, three SARS-CoV-2 mAb therapies have been granted emergency use authorization (EUA) for treatment of non-hospitalized patients with mild-to-moderate COVID-19 in the United States.^4^ However, the high cost of recombinant mAb production and the need for frequent systemic administration pose a major limitation to a broader accessibility. Additionally, none of these mAbs has been approved for prophylactic use.

Over the past several decades, mRNA vaccines have progressed from a skeptical idea to clinical reality with the development of lipid nanoparticle (LNP) deliver system.^5^ mRNA-LNP vaccines have demonstrated as an unprecedented success in response to the emergence of COVID-19 pandemic. Two mRNA vaccines from Pfizer-BioNTech and Moderna have been approved for clinical use, and a panel of other mRNA vaccine candidates are being tested in clinical trials.^6–9^ In addition to vaccine development, the mRNA-LNP platform has been hypothesized as an effective tool for in vivo delivery of any protein of interest for prophylactic and therapeutic purpose. To date, a panel of LNP-encapsulated mRNAs that encode various mAbs have been developed with promising results in preclinical studies to combat viral diseases, including human immunodeficiency virus (HIV), Rabies virus, Influenza virus, Zika virus, Respiratory syncytial virus, and Chikungunya virus (CHIKV).^10–14^ Recently, Moderna finished a phase I clinical trial in the United States (NCT03829384) for an mRNA-based antibody against CHIKV. However, no mRNA antibody against SARS-CoV-2 has been reported yet.

## Results

### In vitro and in vivo characterization of mRNA-HB27-LNP

**Fig. 1:**
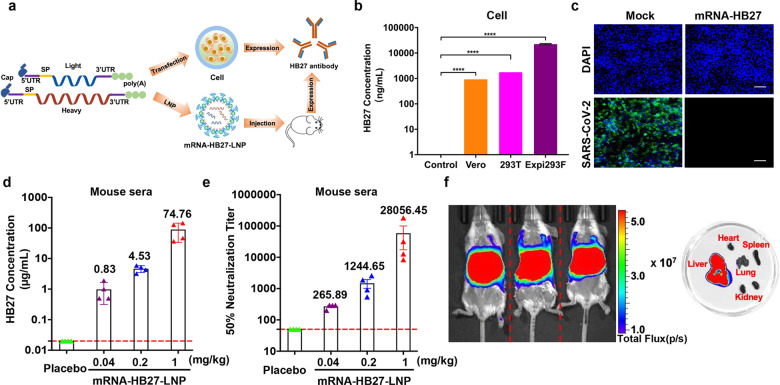
Rational design and characterization of the mRNA antibody. **a** Design and encapsulation of an mRNA antibody mRNA-HB27-LNP. **b** HB27 antibody expression in Vero, 293 T and Expi293F cells at 24 h after transfection with mRNA-HB27 using ELISA assay. Data are shown as mean ± SEM. Data are analyzed by One-way ANOVA with multiple comparisons (****P < 0.0001). **c** Inhibition of SARS-CoV-2 infection in Vero cells at 24 h after transfection with 1 μg of mRNA-HB27 by Immunofluorescence assay. SARS-CoV-2 S protein was stained in green, and DAPI in blue. Scale bars, 100 μm. **d**, **e** The antibody concentration and NT_50_ titer of serum samples in mice. Briefly, female BALB/c mice (*n* = 4/group) were i.v. administrated with the indicated dose of mRNA-HB27-LNP or Placebo. Then, mice sera at 24 h post administration were measured by ELISA (**d**) and SARS-CoV-2 pseudovirus neutralization assay (**e**), respectively. Dotted lines indicate the limits of detection. Data are shown as mean ± SEM. **f** Intravenous injection of reporter mRNA-LNPs for in vivo imaging in mice. IVIS Spectrum image (6 h post-injection) of female BALB/c mice were injected with 10 μg of FLuc-encoding reporter mRNA-LNP by the intravenous route.

Recently, we have developed a potent SARS-CoV-2 neutralizing human mAb HB27, which targets the receptor binding domain (RBD) of spike (S) protein,^15^ and the therapeutic efficacy against mild or moderated COVID-19 patients are being evaluated in phase II clinical trials (NCT04644185). Herein, using the well-established LNP-encapsulated mRNA platform,^16^ we prepared mRNA constructs encoding the HB27 antibody for in vivo delivery. First, the two mRNAs encoding the heavy and light chains of HB27 antibody were constructed (termed as mRNA-HB27) and prepared as previously described (Fig. 1a).^17^ The expression of intact in vitro transcribed (IVT) mRNA-HB27 was determined in multiple cell lines (HEK293T, Vero and Expi293F). The highest production (up to 22,403 ng/mL) was achieved in Expi293F cells (Fig. 1b). SDS-PAGE and western blotting analysis showed that the heavy and light chains expressed by mRNA-HB27 were consistent with original HB27 antibody in protein format (Supplementary information, Fig. S1a, b). Further validation in Vero cells showed that the resulting antibodies from mRNA-HB27 were fully active in neutralizing SARS-CoV-2 (Fig. 1c). These results demonstrated that biological active HB27 antibody can be efficiently produced by mRNAs in vitro.

Then, mRNA-HB27 was processed into the final LNP formulation as previously described,^17^ and the resulting mRNA-encapsulated LNPs were named as mRNA-HB27-LNP. The final stock of mRNA-HB27-LNP showed an average particle size of 88.99 nm (Supplementary information, Fig. S2) and an encapsulation rate of >95%. Then, to assess the in vivo expression profile of mRNA-HB27-LNP, group of BALB/c mice were intravenously (i.v.) administrated with a single dose of mRNA-HB27-LNP at the concentrations of 0.04, 0.2, or 1 mg/kg, respectively. All doses were well tolerated, and no obvious adverse events were observed in all tested mice. Serum antibody concentration and SARS-CoV-2 50% neutralization titer (NT_50_) were determined by ELISA and VSV-based pseudovirus assay,^17^ respectively. As shown in Fig. 1d, e, a dose-dependent production of HB27 antibody and neutralizing activities were detected in mouse sera at 24 h after mRNA-HB27-LNP administration. Especially, the peak serum antibody concentration could reach up to 100 μg/mL, and the NT_50_ geometric mean titer at 1/28,056, and the two readouts showed a great correlation (R2 = 0.9299, *P* < 0.0001) (Supplementary information, Fig. S3). Furthermore, to determine the tissue distribution of our mRNA-LNP preparation following i.v. injection, bioluminescence imaging (BLI) analysis was performed using a firefly luciferase (FLuc) reporter mRNA-LNP.^16^ Robust photon fluxes were readily detected in the abdomen at 6 h post-injection, and further ex vivo imaging clearly demonstrated that liver represented the major target organ (Fig. 1f). Additionally, bio-layer interferometry result indicated that HB27 antibody produced by mRNA-HB27-LNP exhibited higher binding kinetic affinity (*K*D value was 1.56 × 10^−10^ M) for SARS-CoV-2 RBD compared to the results of the original HB27 antibodies produced in CHO cells (*K*D value was 3.09 × 10^−10^ M) (Supplementary information, Fig. S4). These results demonstrated that mRNA-HB27-LNP can efficiently express HB27 antibody following i.v. administration.

### Prophylactically administration of mRNA-HB27-LNP provides a rapid protection against SARS-CoV-2 and its variants in mice and hamsters

**Fig. 2:**
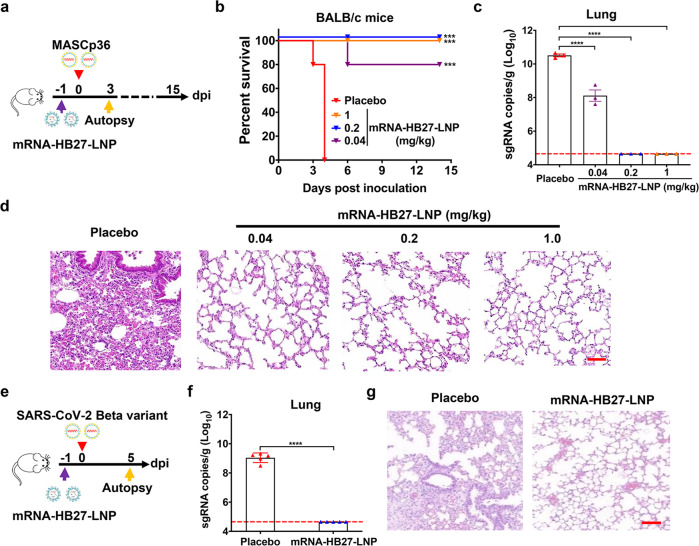
Prophylactic efficacy of mRNA-HB27-LNP against SARS-CoV-2 in mice. **a** Experimental design. Briefly, groups of 8-month-old female BALB/c mice (*n* = 32) were i.v. administrated with a single dose of 1 mg/kg (purple), 0.2 mg/kg (blue), or 0.4 mg/kg (orange) of mRNA-HB27-LNP and Placebo (red) at 1 days before challenge with 6 × 10^3^ PFU of MASCp36, and the clinical symptoms and mortality were recorded for 14 days. Mice (*n* = 3 per group) were sacrificed at 3 dpi for viral detection and histopathological analysis. **b** Survival curves of BALB/c mice administrated with the indicated dose of mRNA-HB27-LNP (*n* = 5 per group) and Placebo (*n* = 5). Data were analyzed by Wilcoxon log-rank survival test. (***P < 0.001, ****P < 0.0001). **c** viral sgRNA loads of lung tissues at 3 dpi were determined by RT-qPCR, respectively. Data are represented as mean ± SEM. Dashed lines represents limit of detection (*n* = 3 per group). Significance was calculated using Ordinary one-way ANOVA Multiple comparisons (****P < 0.0001). **d** Histopathological analysis of lung tissues at 3 dpi. Scale bar, 100 µm. **e** Experimental design. Briefly, the 8-month-old male BALB/c mice received i.v. administration of 1 mg/kg of mRNA-HB27-LNP (*n* = 5) and Placebo (*n* = 5), respectively. At 1 day post administration, mice were i.n. challenged with 1 × 10^4^ PFU of SARS-CoV-2 Beta variant strain, and the lung tissues were collected at 5 days after challenge for detection of viral burden determination (by RT-qPCR) and histopathological analysis. **f** Viral sgRNA loads of lung tissues at 5 dpi were determined by RT-qPCR. Data are represented as mean ± SEM. Significance was calculated using unpaired *t*-test (****P < 0.0001). **g** Histopa*t*hological analysis of lung tissues at 5 dpi. Scale bar, 100 µm.

Next, we sought to assess the in vivo protection efficacy of mRNA-HB27-LNP in the well-established SARS-CoV-2 mouse model using the adapted strain MASCp36.^18^ To this end, groups of 8-month-old BALB/c mice were i.v. administrated with a single dose of different concentrations of mRNA-HB27-LNP, then all animals were intranasally (i.n.) challenged with a lethal dose of MASCp36 at 1 day post mRNA-HB27-LNP administration (Fig. 2a). Survival curve analysis showed that all mice received placebo developed typical respiratory symptoms, and eventually succumbed to severe acute respiratory diseases syndrome (ARDS) within 5 days post MASCp36 challenge. Strikingly, all animals received either 1 mg/kg or 0.2 mg/kg of mRNA-HB27-LNP survived throughout the observation period without any clinical symptoms. The animals received 0.04 mg/kg mRNA-HB27-LNP also achieved 80% survival (Fig. 2b). As expected, high levels of viral sgRNAs (~10^10^ sgRNA copies/g) were detected in lungs from mice received placebo administration. By contrast, the lung viral loads from mice that received 1 mg/kg and 0.2 mg/kg mRNA-HB27-LNP administration were below the detection limit, suggesting the clearance of infectious SARS-CoV-2 in lung tissues (Fig. 2c). Treatment with 0.04 mg/kg of mRNA-HB27-LNP resulted in a ~100-fold reduction (*P* < 0.0001) in viral RNA loads compared with the placebo treatment. More importantly, lung pathology analysis showed that MASCp36 infection caused obvious lung damage characterized by a large area of fused alveoli walls, desquamative epithelial cells, severe edema and scattered hemorrhage in mice of placebo group (Fig. 2d). However, 1 mg/kg and 0.2 mg/kg of mRNA-HB27-LNP treatment completely protected the animals from MASCp36-induced lung damage, and even 0.04 mg/kg of mRNA-HB27-LNP treatment largely alleviated lung damage and only minimal or very mild inflammatory cell infiltration was observed (Fig. 2d).

The emergence and spread of SARS-CoV-2 variants has arose global concerns due to enhanced transmissibility and reduced susceptibility to medical countermeasures.^19^ Therefore, we further evaluated the in vivo protection efficacy of the mRNA-HB27-LNP against the SARS-CoV-2 Beta variant in an established animal model (Fig. 2e).^19^ Briefly, groups of 8-month-old female BALB/c mice received i.v. administration of 1 mg/kg of mRNA-HB27-LNP or placebo, then, all mice were i.n. challenged with 1 × 10^4^ PFU of SARS-CoV-2 Beta variant at 1 day post administration. The lung tissues were collected at 5 days after challenge for detection of viral burden determination (by quantitative reverse transcription PCR (RT-qPCR)) and histopathological analysis. As expected, all mice that received placebo treatment developed high levels of viral sgRNAs (~10^9^ sgRNA copies/g) in lungs; while mRNA-HB27-LNP treatment completely cleared viral sgRNAs in lungs (Fig. 2f). Lung pathology analysis further showed all mice that received placebo administration developed pneumonia around the hilum, characterized with large quantities of desquamative necrotic epithelial cells in bronchioles, a large area of necrotic alveoli pneumocytes and alveoli structure collapse, scattered hemorrhage, inflammatory cells infiltration (Fig. 2g). However, mRNA-HB27-LNP treatment showed a complete protection effect, and no obvious lung damage was seen in all mice (Fig. 2g). These results demonstrated that prophylactically administration of mRNA-HB27-LNP provides rapid protection in mice against SARS-CoV-2 and its variants.

**Fig. 3:**
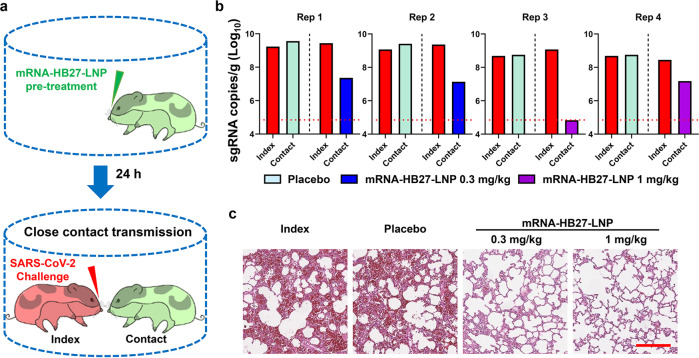
mRNA-HB27-LNP prevents SARS-CoV-2 transmission in hamsters. **a** Experimental design. Briefly, groups of 6–8-week-old female Syrian golden hamsters were i.v. injected with 0.3 or 1 mg/kg of mRNA-HB27-LNP (*n* = 2 per group) or placebo (*n* = 4). 24 h later, each of treatment hamster were transferred to a new cage and were cohoused with one index hamster in a one-to-one manner. The index hamsters (*n* = 8) were infected with 1 × 10^4^ PFU of SARS-CoV-2 through the intranasal route at 1 h prior to cohouse. The lung tissues of hamster were collected at 4 dpi for following-up viral burden determination (by RT-qPCR) and histopathological analysis. **b** Viral sgRNA loads in lung tissues of hamsters at 4 dpi were determined by RT-qPCR. The dotted line in panel shows the limit of detection of the assay. **c** Histopathological analysis of each group of lung tissues at 5 dpi. Representative images from 16 hamsters are shown. Scale bar, 200 µm.

We further evaluated the protection efficacy of mRNA-HB27-LNP in the close contact transmission model of SARS-CoV-2 (Fig. 3a) as previously described.^20^ As shown in Fig. 3b, all contact hamsters that received placebo treatment developed high levels of viral sgRNAs (~10^9^ sgRNA copies/g) in lungs, which is comparable those of donor hamsters. Remarkably, pre-treatment with mRNA-HB27-LNP at 0.3 mg/kg or 1 mg/kg resulted in a sharp reduction in lung viral sgRNA loads, and viral sgRNAs were completely cleared in one animal received high dose pre-treatment. Lung pathology analysis further showed that SARS-CoV-2 caused interstitial pneumonia, with the phenomenon of inflammatory cell infiltration, alveolar septal thickening and distinctive vascular system injury upon donor hamster or contact hamsters that received placebo treatment. In contrast, no obvious lesions were observed in the lung sections from hamster that received mRNA-HB27-LNP treatment (Fig. 3c). These results suggest that prophylactic administration of mRNA-HB27-LNP is sufficient to protect animals in the close contact transmission model.

### mRNA-HB27-LNP possess more excellent pharmacokinetic profile and prophylactic efficacy than HB27

**Fig. 4:**
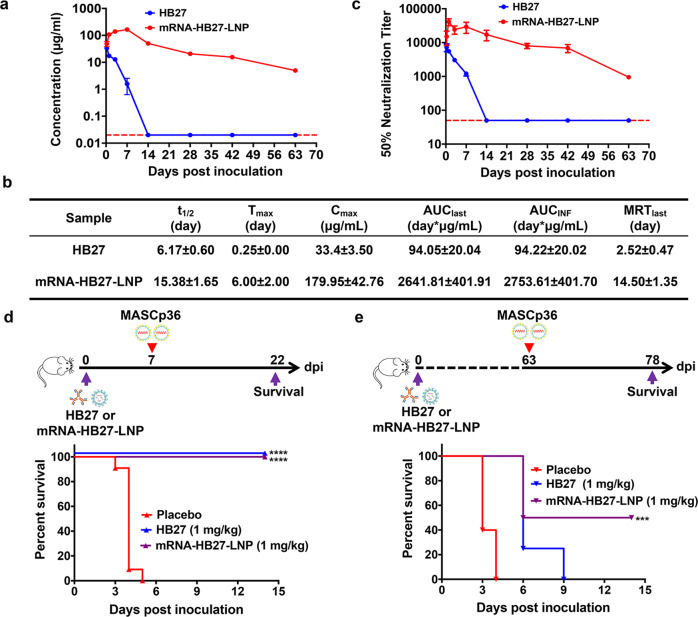
mRNA-HB27-LNP provides a long-term protection against SARS-CoV-2 challenge in mice. **a** The antibody concentration of serum in mice by ELISA. Briefly, groups of 6–8-week-old ICR mice were i.v. administrated with a single dose of 1 mg/kg of HB27 (*n* = 4) and HB27-mRNA-LNP (*n* = 4), respectively. At indicated times post administration, sera of mice were measured by ELISA. Dotted lines indicate the limits of detection. **b** Analysis of antibody pharmacokinetics in serum after the i.v. administration with a single dose of HB27 and mRNA-HB27-LNP. Calculations were performed using WinNolin. **c** NT_50_ of serum in mice by VSV-based SARS-CoV-2 pseudovirus. Data are shown as mean ± SEM. Dashed lines represents limit of detection. **d**, **e** Experimental design. Briefly, groups of 8-month-old female BALB/c mice were i.v. administrated with a single dose of 1 mg /kg of HB27 or mRNA-HB27-LNP (*n* = 4 or 5) and Placebo (*n* = 5). Then at 7 days or 63 days post administration, mice were challenged with 6 × 10^3^ PFU of MASCp36, respectively, and the clinical symptoms and mortality were recorded for 14 days. Survival curves of mice after lethal challenge by MASCp36 at 7 days (**d**) and 63 days (**e**) after the i.v. administration. Data were analyzed by Wilcoxon log-rank survival test (**P < 0.01).

Next, to characterize the pharmacokinetic profile of mRNA-HB27-LNP in mice, 6-week-old female ICR mice (*n* = 4) received a single injection of 1 mg/kg of mRNA-HB27-LNP, or equal dose of original HB27 antibody in protein format, and serum samples were collected at indicated time points, and subjected to antibody concentration and NT_50_ analysis, respectively. As shown in Fig. 4a and b, serum antibody concentration at 6 h after HB27 antibody administration reached to peak with mean value of 33.4 μg/mL, then sharply decreased to the level below detection limit over the next 14 days with the average half-life of 6.17 days. In contrast, mRNA-HB27-LNP administration resulted in higher antibody concentration (48.95 μg/mL) at 6 h post administration, and peaked with mean value of 179.95 μg/mL at 7 days post administration, followed by a gradual decrease. The mean residence time (MRT) and its half-life (t_1/2_) were calculated to 14.5 and 15.38 days, respectively. As a result, the mean AUC_last_ and AUC_inf_ reached 2641.81 and 2753.61 day × μg/mL, respectively (Fig. 4b). Strikingly, the serum antibody concentration maintained 4.95 μg/mL even at 63 days post administration in mice received mRNA-HB27-LNP, and was far beyond the in vitro neutralization concentration 1.47 ng/mL.^15^ Similarly, serum neutralization antibody titers in HB27 antibody-administrated mice peaked at 6 h post administration and decrease sharply below detection limit at day 14 post administration. Surprisingly, serum neutralization antibody titers in mRNA-HB27-LNP treated animals peaked at 24 h post administration, and the NT_50_ geometric mean titer was about 7.0-fold higher (1/37,730 vs 1/5410) compared to the titer from HB27 antibody in protein format (Fig. 4c). Meanwhile, the NT_50_ titer still maintained 1/948 at 63 days post administration (Fig. 4c; Supplementary information, Table S1). This unique pharmacokinetic data suggested a potential long-term protection conferred by mRNA-HB27-LNP.

Based on these results, we further sought to determine the long-term protective efficacy of mRNA-HB27-LNP in comparison with protein antibody. Groups of BALB/c mice were i.v. administered with 1 mg/kg of HB27 or mRNA-HB27-LNP, then i.n. challenge with a lethal dose of MASCp36 at 7 days or 63 days post administration were performed, respectively. As shown in Fig. 4d, e, all mice received placebo administration succumbed to SARS-CoV-2 challenge within 7 days. Strikingly, at 7 days post administration, MASCp36 challenge did not result in any death in mice that received HB27 or mRNA-HB27-LNP, and all mice survived during the observation period (Fig. 4d). Most excitingly, mRNA-HB27-LNP administration protected 50% of mice from SARS-CoV-2 challenge even after 63 days post administration, while all mice that received HB27 or placebo administration died within 9 days (Fig. 4e). These results clearly demonstrated that mRNA-HB27-LNP provide a long-term prophylactic efficacy against lethal SARS-CoV-2 challenge.

## Discussion

The feasibility of in vivo delivery of antibody based on the mRNA-LNP platform has been well demonstrated in recent years, and a dozen of mRNA-encoding mAbs are advancing to clinical trials for prophylactic or therapeutic purpose against infectious diseases or cancers. However, despite the huge need for safe and efficient therapy for COVID-19, mRNA-encoded antibody against SARS-CoV-2 has not been reported to date. Herein, we report the development of the first mRNA antibody against SARS-CoV-2, mRNA-HB27-LNP. The protection efficacy of the original antibody HB27 has been well characterized in several murine models.^15^ Our present data clearly demonstrate that mRNA-HB27-LNP is fully active in preventing SARS-CoV-2 replication and reducing lung damage and mortality in mice, as well as in preventing transmission in hamsters via direct contact (Figs. 2 and 3). These results not only support further development of this unique mRNA antibody into clinical trials, but also validate the potency and versatility of mRNA-LNP technology. At present, thousands of mAbs with potent neutralization activity against SARS-CoV-2 have been characterized,^21,22^ and mRNA constructs encoding these mAbs could be readily prepared in LNP-mRNA formulation for further development. Theoretically, any kind of antibody with biological function can be prepared with this mRNA-LNP platform.

Another unique feature of mRNA-encoded antibody is the increased serum antibody concentration and prolonged circulating half-life. Compared with original HB27 antibody in protein format, the peak serum antibody concentration of mRNA-HB27-LNP is 179.95 μg/mL (about 5.3-fold higher) and the half-life is 15.38 days (about 2-fold longer) (Fig. 4a, b). This feature enables mRNA-HB27-LNP to provide a long-term prophylactic efficacy against lethal SARS-CoV-2 challenge. Previously, Pardi et al.^10^ found that a single i.v. injection of 1.4 mg/kg of mRNA antibody VRC01 against HIV into BALB/c mice resulted in serum antibody concentrations of 170 μg/mL at 24 h post administration, and lasted for up to 9 days. Meanwhile, the dose used for VR01 was much less than that (10 to 20 mg/kg) of antibody in protein format.^10^ Similarly, a single injection of mRNA-LNP encoding mAbs against Rabies virus induced rapid and strong antibody titers in mice, and the half-life was comparable to antibodies in protein format.^23^ Thus, antibodies delivered by mRNA-LNP platform are supposed to confer a much longer protection compared with traditional protein antibodies. The prolonged half-life of mRNA antibodies was probably attributed to the unique in vivo expression profile determined by the LNP formulation, as well as the distinct post-translational modification to traditional antibody manufactured in CHO cells. Indeed, although the mRNA-HB27-LNP is supposed to translate into the same protein antibody HB27 in vivo, the antibody produced from mice injected with mRNA-HB27-LNP showed enhanced binding affinity to SARS-CoV-2 RBD compared to HB27 produced in CHO cells (Supplementary information, Fig. S4).

On the other hand, the continuing emergence and spread of SARS-CoV-2 variant of concerns (VOC) result in a certain degree of resistance to antibodies induced by mRNA vaccine^24^ or neutralizing antibody therapy.^25^ Fortunately, mRNA-HB27-LNP could provide significant protection against the Beta variant challenge in mice (Fig. 4). This protection was probably conferred by intrinsic cross-neutralization capability of HB27, which blocks SARS-CoV-2 not only at attachment to its host cell receptor but also at membrane fusion.^15^ The successful delivery of mRNA encoding a classical mAb like HB27 paved the way to translate other potent antibodies into mRNA-LNP formulations.

In summary, using our well-established mRNA-LNP platform, we prepared LNP-encapsulated mRNA that encodes a known potent SARS-CoV-2 neutralizing mAb, and the resulting mRNA-BH27-LNP at clinical grade is fully capable of producing high level of functional antibody in mice upon i.v. injection. Most importantly, systematic administration of this LNP-mRNA not only induced a sustained circulating antibody with longer half-life than the original antibody in protein format, but also conferred significant protection against SARS-CoV-2 challenge in mouse and hamster models. These promising preclinical results with mRNA-HB27-LNP support further clinical development for the potential application in preventing COVID-19.

## Materials and methods

### Facility and ethics

Experiments involving live SARS-CoV-2 viruses were performed in the biosafety level 3 (BSL-3) facilities in the Beijing Institute of Microbiology and Epidemiology, Academy of Military Medical Sciences (AMMS). All animal experiments were approved by the Experimental Animal Committee of Laboratory Animal Center, AMMS (approval number: IACUC-IME-2021-022).

### Cells and viruses

Vero, 293 T and Expi293F cells were grown in Dulbecco’s modified Eagle’s medium (DMEM) containing 10% (v/v) FBS. SARS-CoV-2 strain Beta CoV/Beijing/IMEBJ01/2020 was originally isolated from a COVID-19 patient from Wuhan, China.^17^ Mouse adapted strain of SARS-CoV-2 (MASCp36) was developed in our previous study. The SARS-CoV-2 Beta variant was isolated from an imported patient from South Africa and Stored at National Pathogen Resource Center (NPRC), China.^19^ These viruses were amplified and titrated by standard plaque forming assay on Vero cells. The production and purification of HB27 in CHO cells were performed by SinoCell Tech.^15^

### mRNA preparation and LNP formulation

The plasmids HB27-H and HB27-L encoding the codon-optimized heavy and light chain region of HB27 antibody were synthesized by GENEWIZ, and confirmed by DNA sequencing. The mRNA was produced in vitro using T7 RNA polymerase-mediated transcription from the linearized DNA template from plasmids HB27-H and HB27-L, respectively. The final pseudouridine-modified mRNAs contained a 5′ cap (Cap1), a 5′ UTR consisting of a partial sequence of the xenopus laevis Beta globin gene, a coding region of HB27-H or HB27-L, a 3′ UTR consisting of a partial sequence of the Homo sapiens hemoglobin subunit alpha gene, and a poly (A) tail. The final mRNA-LNP formulations were prepared using a modified procedure of a method previously described for mRNA vaccine,^17^ and the mRNA concentration, purity, and encapsulation were assayed for quality control. All procedures were carried out in GMP condition.

### The in vitro expression of mRNA-HB27

Vero, 293 T, Expi293F cells were seeded in 24-well plates at 200,000 cells/well. Eighteen hours later, the cells were transfected with mRNA-HB27 using Lipofectamine™ 3000 Transfection Reagent (Thermo Fisher Scientific). Six hours later, the medium was replaced with Opti-MEM™ I Reduced Serum Medium (Thermo Fisher Scientific). The supernatant was collected at 48 h after transfection and analyzed by ELISA as described below.

### The in vivo expression dynamics of mRNA-HB27-LNP

Female BALB/c mice (8-month-old) were purchased from Beijing Vital River Laboratory Animal Technology Co., Ltd. (Beijing, China). Sixteen mice were randomly divided into four groups (*n* = 4/group). HB27 antibody or mRNA-HB27-LNP or LNP was i.v. administrated at 0.04 mg/kg, 0.2 mg/kg, or 1 mg/kg into mice. The orbital blood was collected at 24 h after administration, centrifuged at 5000 × *g* at 4 °C for 10 min. Sera were collected and stored at −80 °C for further test. Antibody expression level was determined by ELISA and pseudovirus neutralization assay as described below.

Female ICR mice (6-week-old) were purchased from Shanghai SLAC Laboratory Animal Co., Ltd. (Shanghai, China). And randomly divided into two groups (*n* = 4/group). HB27 antibody or mRNA- HB27-LNP was intravenously administrated at 1.0 mg/kg into animals. The orbital blood was collected at 0.25, 1, 3, 7, 14, 28, 42 and 63 days after administration, centrifuged at 5000 × *g* at 4 °C for 10 min. Sera were collected and stored at −80 °C for further test. Serum antibody concentration and NT_50_ were determined by ELISA and SARS-CoV-2 pseudovirus neutralization assay, respectively. Then these data were performed using WinNolin.

### ELISA assay

Evaluation of HB27 expression in vitro and in vivo was performed by ELISA as described previously.^15^ Briefly, 96-well microtiter plates were coated with the recombinant RBD protein (Sino Biological) overnight at 4 °C. The coated plates were washed once with PBS and blocked with 5% BSA at 37 °C for 1 h. Plates were then washed twice with PBS and incubated with calibrators and serial dilutions of cell culture media or mouse sera at 37 °C for 1 h, prior to three further washes and subsequent 1 h incubation with HRP-conjugated goat anti-human IgG-Fc antibody (Biodragon) as secondary antibody, followed by incubation with TMB substrate (Solarbio). The absorbance at 450/620 nm was measured and accurate quantification were conducted using SpectraMax iD3 (Molecular Devices). Each quantitative test produces a standard curve for back-calculation of accurate concentration of HB27.

### Pseudovirus neutralization assay

The SARS-CoV-2 pseudovirus neutralization assay was performed as described previously.^17^ Briefly, Huh7 cells were seeded in 96-well plates (30,000 cells/well) and incubated for approximately 24 h until 90%–100% confluent. Then, serial diluted serum samples were incubated with SARS-CoV-2 pseudovirus for 1 h at 37 °C and were added into the wells followed by incubation for 20–24 h in a 5% CO_2_ environment at 37 °C. Finally, the luciferase luminescence (RLU) was measured using luciferase assay system following the manufacturer’s manual with a luminescence microplate reader. The neutralization percentage was calculated using the formula: Inhibition (%) = [1 – (sample RLU–Blank RLU)/(Positive Control RLU–Blank RLU)] (%). Neutralization titers were presented as 50% maximal inhibitory concentration (NT_50_).

### Immunofluorescent assay

Briefly, Vero cells were transfected with different concentration of mRNA-HB27 using Lipofectamine™ 3000 Transfection Reagent (Thermo Fisher Scientific). At 24 h later, cells were infected with approximately 100 PFU of SARS-CoV-2 and incubated at 37 °C for 60 min. Then, the mixture was removed, and 1 mL of DMEM plus 4% (v/v) FBS with 2% LMP agarose was added onto the infected cells. After further incubation at 37 °C for 48 h, the wells were fixed with 4% paraformaldehyde (PFA) for 20 min at room temperature and stained RBD protein with and for nuclei with 4,6-diamidino-2-phenylindole (DAPI) in turn. The fluorescence images were recorded using a microscope.

### Prophylactic efficacy of mRNA-HB27-LNP in mice

The in vivo prophylactic efficacy of mRNA-HB27-LNP was assessed by using the well-established mouse model based on a SARS-CoV-2 mouse adapted strain MASCp36^17^ and Beta variant strain,^19^ respectively. For MASCp36 model, groups of 8-month-old female BALB/c mice (*n* = 32) were i.v. administrated with different doses of mRNA-HB27-LNP (0.04, 0.2 and 1 mg/kg) or empty LNP was used as placebo. At 24 h before challenge with 6 × 10^3^ PFU of MASCp36 via the i.n. route. All mice were monitored daily for morbidity and mortality. The lung tissues of mice were collected at 3 day post infection (dpi) for following-up viral burden determination (by RT-qPCR) and pathological examination.

The in vivo long-term protective effect of HB27 or mRNA-HB27-LNP was evaluated using the SARS-CoV-2 mouse-adapted strain (MASCp36) animal model as described previously. Briefly, a group of 8-month-old female BALB/c mice were i.v. administrated of 1 mg/kg of HB27 (*n* = 8) or mRNA-HB27-LNP (*n* = 8), empty LNP (*n* = 10) was used as placebo. Mice were i.n. challenged with a lethal dose of 6×10^3^ PFU of MASCp36 at 7 days or 63 days after administration. All mice were monitored daily for morbidity and mortality for 14 days after infection.

For the SARS-CoV-2 Beta variant strain model, groups of 8-month-old female BALB/c mice (*n* = 10) received i.v. administration of 1 mg/kg of mRNA-HB27-LNP, and empty LNP was used as placebo. At 1 day post administration, mice were i.n. challenged with 1 × 10^4^ PFU of SARS-CoV-2 Beta variant, and the lung tissues were collected at 5 days after challenge for detection of viral burden and pathological examination.

### Prophylactic efficacy of mRNA-HB27-LNP in hamsters

The prophylactic effect of mRNA-HB27-LNP were evaluated by using the well-established Syrian golden hamster model.^20^ In brief, each 6–8-week-old female Syrian golden hamster pre-treated with 0.3 or 1 mg/kg of mRNA-HB27-LNP via the i.v. route was transferred to a new cage, and co-housed with one index hamster in a one-to-one manner. The index hamster was i.n. infected with 1 × 10^4^ PFU of SARS-CoV-2 at 1 h prior to cohouse. The lung tissues of hamsters were collected at 4 dpi for viral burden determination (by RT-qPCR) and pathological examination.

### Viral burden determination

Viral burden in lung from mice and hamster were measured as described previously.^19^ Briefly, lung tissue homogenates were clarified by centrifugation at 6000 rpm for 6 min and viral RNA was extracted using the QIAamp Viral RNA Mini Kit (Qiagen) according to the manufacturer’s protocol. Viral sgRNA quantification in each tissue sample was performed by RT-qPCR targeting the S gene of SARS-CoV-2. RT-qPCR was performed using One-Step PrimeScript RT-PCR Kit (Takara). The primers and probes used for viral sgRNA quantification were sgRNA-F (5′-CGATCTCTTGTAGATCTGTTCTC-3′); sgRNA-R (5′-ATATTGCAGCAGTACGCACACA-3′); and sgRNA-P3 (5′-ACACTAGCCATCCTTACTGCGCTTCG-3′).

### Lung histology

Lung tissues from mice were fixed with perfusion fixative (formaldehyde) for 48 h, and embedded in paraffin according to standard histological assays. Then, lung tissues were stained with hematoxylin and eosin (H&E). Images were captured using Olympus BX51 microscope equipped with a DP72 camera.

### Bioluminescence imaging

To detect the tissue distribution of mRNA-LNP formulation upon intravenous administration in mice, an FLuc reporter mRNA-LNPs was used for BLI as described previously.^16^ Briefly, 6–8-week-old female BALB/c mice (*n* = 3) were inoculated with 10 μg of FLuc mRNA-LNP via the i.v. route. Six hours after injection, animals were given an intraperitoneal injection of luciferase substrate (Promega), and fluorescent signals were collected for 60 s with an IVIS Spectrum instrument (PerkinElmer). The heart, liver, spleen, lung, and kidney tissues were collected, and the fluorescence signal of each tissue was detected for 60 s. The fluorescence signal of the region of interest (ROI) was quantified using Living Image 3.0.

### Statistical analysis

All data were analyzed with GraphPad Prism 9.0 software. Unless specified, data are presented as mean ± SEM in all experiments. The statistical significance was assessed by One-way ANOVA with multiple comparisons or unpaired *t*-test while in survival test, the statistical significance was analyzed by Wilcoxon log-rank. (*P < 0.05; **P < 0.01; ***P < 0.001; ****P < 0.0001; n.s., not significant).

## Supplementary information

Supplementary materials and methods

Supplementary information Fig. S1

Supplementary information Fig. S2

Supplementary information Fig. S3

Supplementary information Fig. S4

Supplementary information Table S1
