# Supplementary material for: Lipid nanoparticle-encapsulated mRNA antibody provides long-term protection against SARS-CoV-2 in mice and hamsters
Source: Cell Res. 2022 Feb 24;32(4):375–82. doi: 10.1038/s41422-022-00630-0 (PMC8866932; doi:10.1038/s41422-022-00630-0)
Supplement: Supplementary file 1 — Supplementary materials and methods [file 41422_2022_630_MOESM1_ESM.pdf]

Supplementary Materials for

Lipid nanoparticle-encapsulated mRNA antibody provides long-term protection against SARS-CoV-2 in mice and hamsters

Yong-Qiang Deng[1], Na-Na Zhang[1,2], Yi-Fei Zhang[1,3], Xia Zhong[4], Sue Xu[4], Hong-Ying Qiu[1], Tie-Cheng Wang[5], Hui Zhao[1], Chao Zhou[1], Shu-Long Zu[1], Qi Chen[1], Tian-Shu Cao[1], Qing Ye[1], Hang Chi[1], Xiang-Hui Duan[4], Dan-Dan Lin[4], Xiao-Jing Zhang[4], Liang-Zhi Xie[6], Yu-Wei Gao[5], Bo Ying[4*], Cheng-Feng Qin[1,2*]

1. State Key Laboratory of Pathogen and Biosecurity, Beijing Institute of Microbiology and Epidemiology, AMMS, Beijing, China

2. School of Medicine, Tsinghua University, Beijing, China

3. Kunming University of Science and Technology, Kunming, Yunnan, China.

4. Suzhou Abogen Biosciences Co., Ltd., Suzhou, Jiangsu, China.

5. Key laboratory of Jilin Province for Zoonosis Prevention and Control, Changchun, Jilin, China

6. Beijing Protein and Antibody R&D Engineering Center, Sinocelltech Ltd., Beijing, China

These authors contributed equally: Yong-Qiang Deng Author, Na-Na Zhang Author, Yi-Fei Zhang Author, Xia Zhong Author, Sue Xu Author, Hong-Ying Qiu Author

Correspondence: Bo Ying (bo.ying@abogenbio.com) or Cheng-Feng Qin (qincf@bmi.ac.cn)

Materials and Methods

SDS-PAGE and Western Blotting

293T cell were seeded in T175 cell culture flasks at 10^6 cells/flasks. Eighteen hours later, the cells were transfected with equal molar of Heavy and Light chain of original HB27 antibody or mRNA-HB27 using Lipofectamine™ 3000 Transfection Reagent (Thermo Fisher Scientific). After 6 h, the medium was replaced with Opti-MEM™ I Reduced Serum Medium (Thermo Fisher Scientific). The supernatant was collected at 48 h after transfection, clarified by centrifugation at 1000 ×g, and then mixed with 5×SDS loading buffer. The samples were loaded for SDS-PAGE. The secreted HB27 protein was then detected by Western blotting with anti-human IgG-Fc/HRP and goat anti-human IgG F(ab')2/HRP antibody (Thermo Fisher Scientific).

Bio-layer interferometry

The binding kinetic affinity between SARS-CoV-2 RBD-Fc and HB27 was measured by gator (Probe life). Recombinant SARS-CoV-2 RBD-Fc protein (100 nM) was captured by anti-HIgG Fc probes (Probe life). Then probes were individually inserted into buffer (0.2% IgG free BSA and 0.01% tween20 in PBS) containing HB27 at different concentrations (from 12.5 nM to 0.8 nM) for association and then inserted into buffer (0.2% IgG free BSA and 0.01% tween20 in PBS) for disassociation. The wave shift was analyzed by gator software and the data were analyzed by Prism.
