# Supplementary material for: Lipid nanoparticle-encapsulated mRNA antibody provides long-term protection against SARS-CoV-2 in mice and hamsters
Source: Cell Res. 2022 Feb 24;32(4):375–82. doi: 10.1038/s41422-022-00630-0 (PMC8866932; doi:10.1038/s41422-022-00630-0)
Supplement: Supplementary file 2 — Supplementary information Fig. S1 [file 41422_2022_630_MOESM2_ESM.pdf]

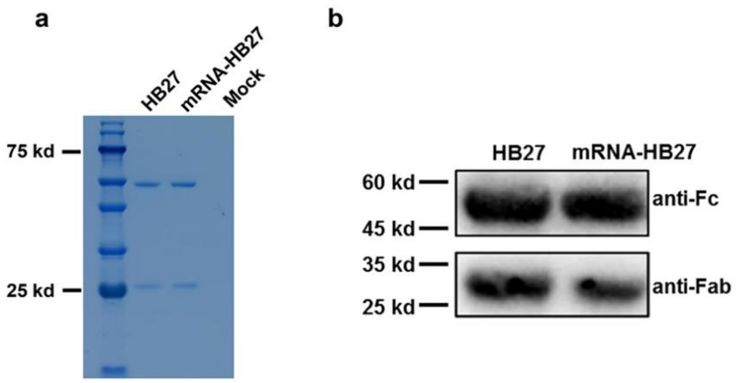

Fig. S1. Characterization of mRNA antibody in vitro. Related to Fig. 1.

Briefly, 293T cells were transfected with 10 μg of mRNA-HB27. At 24 h after transfection, expression of light and heavy chain of mRNA-HB27 antibody in supernatant were performed by SDS-PAGE (a) and western blotting analysis (b) with anti-human IgG-Fc/HRP and goat anti-human IgG F(ab')2/HRP antibody, respectively.
