# Supplementary material for: Lipid nanoparticle-encapsulated mRNA antibody provides long-term protection against SARS-CoV-2 in mice and hamsters
Source: Cell Res. 2022 Feb 24;32(4):375–82. doi: 10.1038/s41422-022-00630-0 (PMC8866932; doi:10.1038/s41422-022-00630-0)
Supplement: Supplementary file 3 — Supplementary information Fig. S2 [file 41422_2022_630_MOESM3_ESM.pdf]

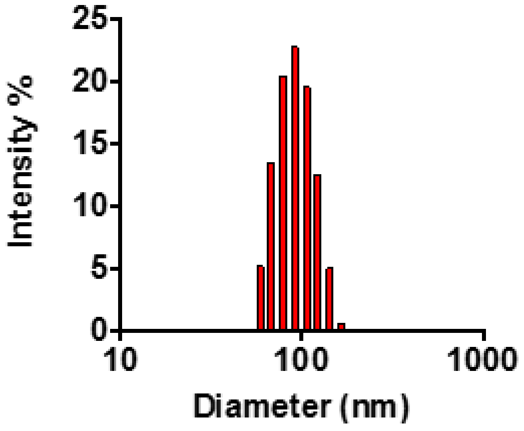

Fig. S2. Representative intensity-size graph of mRNA-HB27-LNP measured by dynamic light scattering method. Related to Fig. 1.
