# Supplementary material for: Lipid nanoparticle-encapsulated mRNA antibody provides long-term protection against SARS-CoV-2 in mice and hamsters
Source: Cell Res. 2022 Feb 24;32(4):375–82. doi: 10.1038/s41422-022-00630-0 (PMC8866932; doi:10.1038/s41422-022-00630-0)
Supplement: Supplementary file 4 — Supplementary information Fig. S3 [file 41422_2022_630_MOESM4_ESM.pdf]

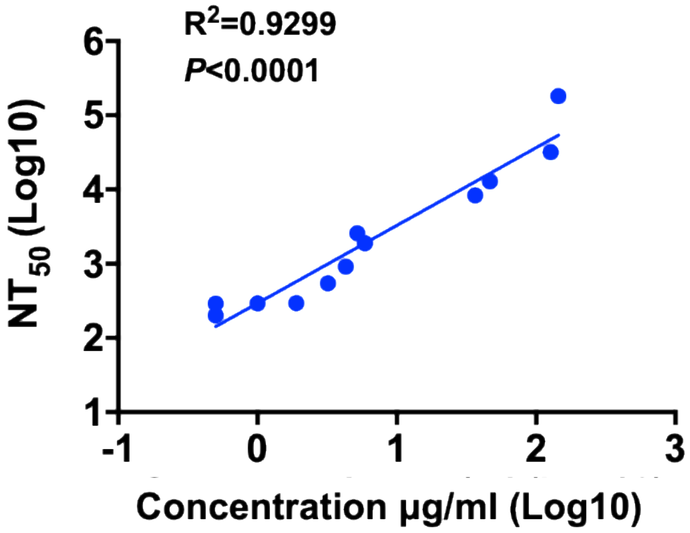

Fig. S3. Correlations of serum antibody concentration and SARS-CoV-2 neutralization titer (NT_{50}). Animals receiving a mRAN-HB27-LNP (n=12) injection were included in this analysis. The *P* values and R^2 values reflect Spearman rank-correlation tests. Related to Fig. 1.
