# Supplementary material for: Lipid nanoparticle-encapsulated mRNA antibody provides long-term protection against SARS-CoV-2 in mice and hamsters
Source: Cell Res. 2022 Feb 24;32(4):375–82. doi: 10.1038/s41422-022-00630-0 (PMC8866932; doi:10.1038/s41422-022-00630-0)
Supplement: Supplementary file 5 — Supplementary information Fig. S4 [file 41422_2022_630_MOESM5_ESM.pdf]

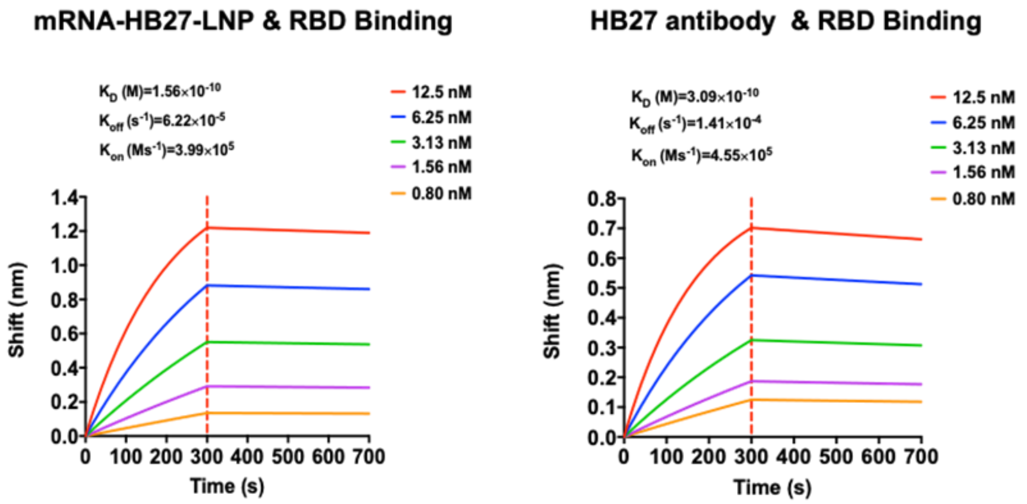

Fig. S4. Affinity Analysis of HB27 and mRNA-HB27-LNP for SARS-CoV-2 RBD by bio-layer interferometry. Related to Fig. 1.

Briefly, BALB/c mice were i.v. administrated of HB27 antibody and mRNA-HB27-LNP. Then, serum was collected at 1 day post administration and immobilized onto the sensing probes coated with RBD and tested for real-time association and dissociation of the HB27 antibody. The light wave shifts were recorded and curves were fitted by gator (Probe life).
