# Supplementary material for: Lipid nanoparticle-encapsulated mRNA antibody provides long-term protection against SARS-CoV-2 in mice and hamsters
Source: Cell Res. 2022 Feb 24;32(4):375–82. doi: 10.1038/s41422-022-00630-0 (PMC8866932; doi:10.1038/s41422-022-00630-0)
Supplement: Supplementary file 6 — Supplementary information Table S1 [file 41422_2022_630_MOESM6_ESM.pdf]

**Table S1. Analysis of $NT_{50}$ of serum in mice after the i.v. administration with a single dose of 1 mg/kg of HB27 and mRNA-HB27-LNP.**

Calculations were performed using WinNolin.

| Antibody | $t_{1/2}$ (day) | $T_{max}$ (day) | $C_{max}$ ($NT_{50}$) | $AUC_{last}$ (day*$NT_{50}$) | $AUC_{INF}$ (day*$NT_{50}$) | $MRT_{last}$ (day) |
|---|---|---|---|---|---|---|
| **HB27** | **9.06±0.77** | **0.93±0.43** | **7821.25±3572.96** | **30699.00±5057.04** | **31352.54±5017.47** | **5.91±0.68** |
| **mRNA-HB27-LNP** | **13.45±4.57** | **4.25±6.50** | **43590.25±16114.20** | **720997.02±239621.22** | **738726.75±237161.03** | **18.43±3.83** |
